# A bibliometric analysis of the cannabis and cannabinoid research literature

**DOI:** 10.1186/s42238-022-00133-0

**Published:** 2022-05-25

**Authors:** Jeremy Y. Ng, Nathan Chang

**Affiliations:** Michael G. DeGroote Centre for Learning and Discovery, Department of Health Research Methods, Evidence, and Impact, Faculty of Health Sciences, McMaster University, Room 2112, 1280 Main Street West, Hamilton, Ontario L8S 4K1 Canada

**Keywords:** Bibliometric analysis, Cannabis, Marijuana, Research trends, Scientometrics

## Abstract

**Background:**

Cannabis refers to a plant in the family Cannabaceae, which has been used medically, recreationally, and industrially. The last two decades, in particular, have seen a large increase in the volume of literature on this topic. The present bibliometric analysis aims to capture the characteristics of scholarly journal publications on the topic of cannabis and cannabinoid research.

**Methods:**

Searches were run on the Scopus database on April 02, 2021, as follows “(TITLE (cannabi* OR hashish OR marijuana OR marihuana)) AND ( LIMIT-TO ( DOCTYPE,"ar" ) OR LIMIT-TO ( DOCTYPE,"re" ) )”. Results were exported on the same day to prevent discrepancies between daily database updates. Only “article” and “review” publication types were included; no further search limits were applied. The “article” publication type includes publications featuring original research, whereas “review” includes reviews and conference papers. The following data were collected: number of publications (in total and per year), authors, and journals; open access status; publications per journal; journals publishing the highest volume of literature and their impact factors, language of publication; document type; publication country; author affiliations; funding sponsors; most highly cited publications; and most highly published authors. Trends in this subset of publications were identified and presented. Bibliometric networks were constructed using the software tool VOSviewer.

**Results:**

A total of 29 802 publications (10 214 open access), published by 65 109 authors, were published in 5474 journals from 1829 to 2021. The greatest number of publications was published over the last 20 years. The journal that published the largest number of publications was Drug and Alcohol Dependence (*n* = 705). The most productive countries included the USA (*n* = 12 420), the UK (*n* = 2236), and Canada (*n* = 2062); many of the most common institutional affiliations and funding sponsors originated from these countries.

**Conclusions:**

The number of publications published on the topic of cannabis follows an upward trend. Over the past 20 years, the volume of cannabis research has grown steeply, which can be attributed to a large amount of funding dedicated to researching this topic. Future research should continue to investigate changes in the publication characteristics of emerging research, as the volume of publications on this topic is expected to rapidly grow.

**Supplementary Information:**

The online version contains supplementary material available at 10.1186/s42238-022-00133-0.

## Background

Cannabis refers to a flowering plant in the family Cannabaceae; while the exact number of species within the genus is disputed, the following three are generally recognized: *Cannabis sativa*, *Cannabis indica*, and *Cannabis ruderalis*. The cannabis plant contains about 540 chemical substances (National Center for Complementary and Integrative Health (NCCIH) 2019), and over 100 of them are classified as cannabinoids, of which cannabidiol (CBD) and tetrahydrocannabinol (THC) are the most prominent. THC is primarily responsible for the plant’s psychoactive effects (National Center for Complementary and Integrative Health (NCCIH) 2019; Health Canada 2018), while CBD is of particular interest to healthcare researchers and clinicians, as this specific compound is responsible for cannabis’ purported therapeutic value (Harvard Health Publishing 2018). The term “marijuana” refers to the parts of or the products from the cannabis plant that contain THC, while industrial hemp refers to plants that have minimal THC (National Center for Complementary and Integrative Health (NCCIH) 2019). Cannabis is sometimes used interchangeably with the term “hemp”; however, the latter only refers to varieties cultivated for non-drug uses. The cannabis plant is widely used for hemp fiber, hemp seeds and oils, hemp leaves for use as vegetables and juices, and as a recreational drug (Small 2015). Medically, cannabis has long been considered of value as a general analgesic, anesthetic, antidepressant, antibiotic, and sedative; its history dates back to 2700 BCE when cannabis was first documented in a Chinese pharmacopeia by Chinese Emperor Shen Nung, who is widely regarded as the Father of Chinese Medicine (Touw 1981; Pisanti and Bifulco 2019).

The effects of cannabis are dependent on strength and quantity, the environment in which it is taken, and the experience of the individual using it. Psychological effects tend to predominate, with the user commonly experiencing mild euphoria. Individuals using cannabis often report distortions in time and space (National Academies of Sciences, Engineering, and Medicine n.d.). Acute intoxication may result in the following: visual hallucinations, anxiety, depression, extreme variability of mood, paranoid reactions, and psychoses lasting up to six hours. Physical effects include reddening of the eyes, mouth and throat dryness, moderate increases in heartbeat, tightness in the chest (if smoked), drowsiness, unsteadiness, and muscular incoordination (D’Souza et al. 2004; Ashton 1999; Karila et al. 2014). Many questions concerning the medical and social impacts of individuals using cannabis globally have been of interest to researchers since THC was first synthesized and isolated in 1969 (Mechoulam and Burstein 1973). Cannabinoids are now known to affect cell receptors in the brain and body, changing how they behave and communicate (Health Canada 2018), and may serve as a promising therapy in treating and/or managing epilepsy, nausea, and vomiting induced by cancer chemotherapy and weight loss, loss of appetite associated with HIV AIDS, chronic pain, and muscle spasticity associated with multiple sclerosis (National Center for Complementary and Integrative Health (NCCIH) 2019).

The last few decades have seen a large increase in the volume of literature on the topic of cannabis and cannabinoids (Liu et al. 2020; Matielo et al. 2018), and the application of a bibliometric analysis can facilitate a stronger understanding of the field. Most recently, extensive cannabis research efforts have taken place in countries such as Canada, where on October 17, 2018, the Cannabis Act came into force, legalizing the sale and use of recreational cannabis across Canada (Government of Canada 2018), also making the substance easier to study. A bibliometric analysis is a research methodology that involves the statistical assessment of scientific publications or books, to identify the characteristics and determine the impact of the literature published in a specific academic discipline (Pritchard 1969; Price 1976; Hicks et al. 2015).

### Comparative literature

To date, only a small number of bibliometric analyses on the topic of cannabis and cannabinoid research have been conducted. Liu et al. (2020) conducted a bibliometric analysis of cannabis and cannabidiol research published between 1940 and 2019, capturing the characteristics of 1167 publications. They found that the historical development of this research topic could be divided into studies that focused on the following three aspects: chemistry, pharmacology, and molecular biology (Liu et al. 2020). Another bibliometric analysis conducted by Matielo et al. (2018) captured the characteristics of six decades of research on the cannabis plant totaling 1284 publications. This number does not reflect the entirety of the cannabis and cannabinoid literature given they sought to capture research conducted at the intersection of cannabis and the following six topics only: biochemical, biology, forensic genetics, genetics, molecular markers, and traceability (Matielo et al. 2018). Prior to this study, the two aforementioned bibliometric analyses represented the most comprehensive ones to date with respect to cannabis and cannabinoid literature. Others have focused on more specific subsets of publications; for example, Yeung et al. (2019) analyzed the 100 most highly cited studies published on the topic of cannabis, cannabinoids, and endocannabinoids (Yeung et al. 2019). Treister-Goltzman et al. (2019) identified trends in publications specific to medical cannabis; the authors found a large increase in the volume of publications on this topic over the approximate last two decades, much of which originated from the USA (Treister-Goltzman et al. 2019). Lastly, three additional bibliometric analyses are worth mentioning as they all sought to characterize a subset of publications relating to substance use and addictions research, all of which included cannabis (Zurián et al. 2021; Sweileh et al. 2014; Bramness et al. 2014).

### Study objective

The objective of the present bibliometric analysis is to capture the characteristics of scholarly journal publications on the topic of cannabis and cannabinoid research, highlight potential areas of growth, and understand what new knowledge is being created.

## Methods

Bibliometric analysis is a method commonly used to explore the development of research in a particular field (Donthu et al. 2021). It relies on the quantitative analysis of large quantities of data to describe large scope trends, such as journal performance and the demographics of contributions.

Large datasets can be obtained through searches of databases (Donthu et al. 2021), such as Scopus, as used in the present study. The ensuing analysis can benefit a variety of inquiries by analyzing the performance of research in a field using publication-based metrics, such as the number of contributions and productivity, or citation-based metrics, such as total and average citations (Donthu et al. 2021). Performance-based analysis can be used to depict trends in a field, such as the productivity and impact of authors and journals (Donthu et al. 2021). Bibliometric analysis may also be used to map out the interactions between contributors in a field through methods such as citation analysis and co-occurrence analysis of keywords (Donthu et al. 2021). Both performance-based analysis and mapping were used in the present study to examine potential trends in the field of cannabis and cannabinoid research by analyzing the number of contributions per year, analyzing the demographics of contributions, and understanding the interactions of contributions by identifying the most influential publications. Network analysis may be used to further understand the relationships between contributors and publications (Van Eck and Waltman 2014). This may reveal information about the prevalence of certain countries, authors, and journals within the field. VOSviewer is a software that generates network visualization maps, along with other types of bibliometric webs, to facilitate this form of analysis. Each network visualization web is constructed of nodes containing types of data (e.g., keywords, countries of publication, etc.) that are connected by edges to show an interaction between two given nodes (Van Eck and Waltman 2014). The generated web utilizes the 2-dimensional distance between nodes to depict the strength of their interaction (McAllister et al. 2021; Van Eck and Waltman 2010). Additionally, highly related nodes are grouped into clusters of a specific color to facilitate the categorization of data and relationships (Van Eck and Waltman 2014; McAllister et al. 2021; Van Eck and Waltman 2010). The present study utilizes network visualization webs to elucidate the interactions between cannabis research and other areas of research.

### Publication search and characteristics

A single search was run on Scopus on April 02, 2021, as follows: “(TITLE (cannabi* OR hashish OR marijuana OR marihuana)) AND ( LIMIT-TO ( DOCTYPE,“ar” ) OR LIMIT-TO ( DOCTYPE,"re" ) )”. Search results were exported in batches due to the download limits imposed by Scopus; all downloads were completed on the same day to prevent discrepancies between daily database updates. Searches were only conducted on Scopus because it is the largest abstract and citation database of scholarly literature (Elsevier n.d.); in comparison, Web of Science contains considerably fewer indexed publications, while OVID databases do not provide certain metrics such as publication citation counts (Gusenbauer and Haddaway 2020). Only “article” and “review” publication types were included, and no further search limits were applied. The “article” publication type includes publications featuring original research or opinions, whereas the “review” publication type includes reviews of original research and conference papers (Elsevier n.d.). The following bibliometric data were collected: number of publications (in total and per year), authors, and journals; open access status; publications per journal; journals publishing the highest volume of literature and their impact factors, language of publication; document type; publication country; author affiliations; funding sponsors; most highly cited publications; and most highly published authors. Publications counted towards an author’s total, regardless of the order of authorship. Trends associated with this subset of publications were identified and presented. Bibliometric networks were constructed and visualized using the software tool VOSviewer (version 1.6.1) (Van Eck and Waltman 2010; Van Eck and Waltman 2020).

### Citation analysis

Citation analysis uses the number of citations a given research item receives to quantify its relative impact within a field of research (Donthu et al. 2021). A citation analysis of journals that published the largest volume of cannabis and cannabinoid research was performed to identify possible trends between a journal’s volume of publication and its relative impact through the number of citations. The impact of each of the 30 highest-producing journals was quantified using the 2019 journal impact factor, calculated as the ratio between the number of citations to research published by a journal in the previous 2 years and the number of citable research (Journal impact factor (2021)). Each of the 30 journals containing the highest number of publications was hand-searched on InCites Journal Citation Reports to obtain the 2019 journal impact factor. A bibliometric network was constructed and visualized using the software tool VOSviewer (version 1.6.1) (Van Eck and Waltman 2010; Van Eck and Waltman 2020).

### Co-occurrence analysis of keywords

Keyword co-occurrence analysis is used to identify relationships between the contents of multiple research items by examining the use of common keywords to summarize research (Donthu et al. 2021). A co-occurrence analysis of the data derived from Scopus was used to find the most common author keywords used in publications. This was used to map out promising areas of study in cannabis and cannabinoid research by exploring its intersectionality with other topics and fields. A bibliometric network was constructed and visualized using the software tool VOSviewer (version 1.6.1) (Van Eck and Waltman 2010; Van Eck and Waltman 2020).

### Co-authorship analysis

Co-authorship analysis examines formalized collaborations to understand how researchers in a specific field interact with others (Donthu et al. 2021). A co-authorship analysis of the countries that published the highest volume of cannabis and cannabinoid research was used to determine patterns of collaboration between authors from two different countries. A bibliometric network was constructed and visualized using the software tool VOSviewer (version 1.6.1) (Van Eck and Waltman 2010; Van Eck and Waltman 2020).

## Results

**Fig. 1:**
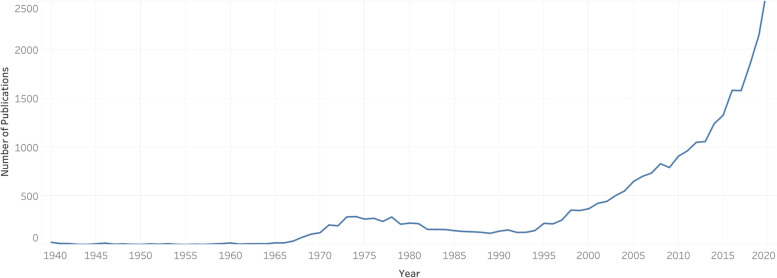
Number of cannabis and cannabinoid publications per year from 1940 to 2020. Data was collected from Scopus in a single search. The year 2021 was omitted due to the year being incomplete at the time the search was conducted

**Fig. 2:**
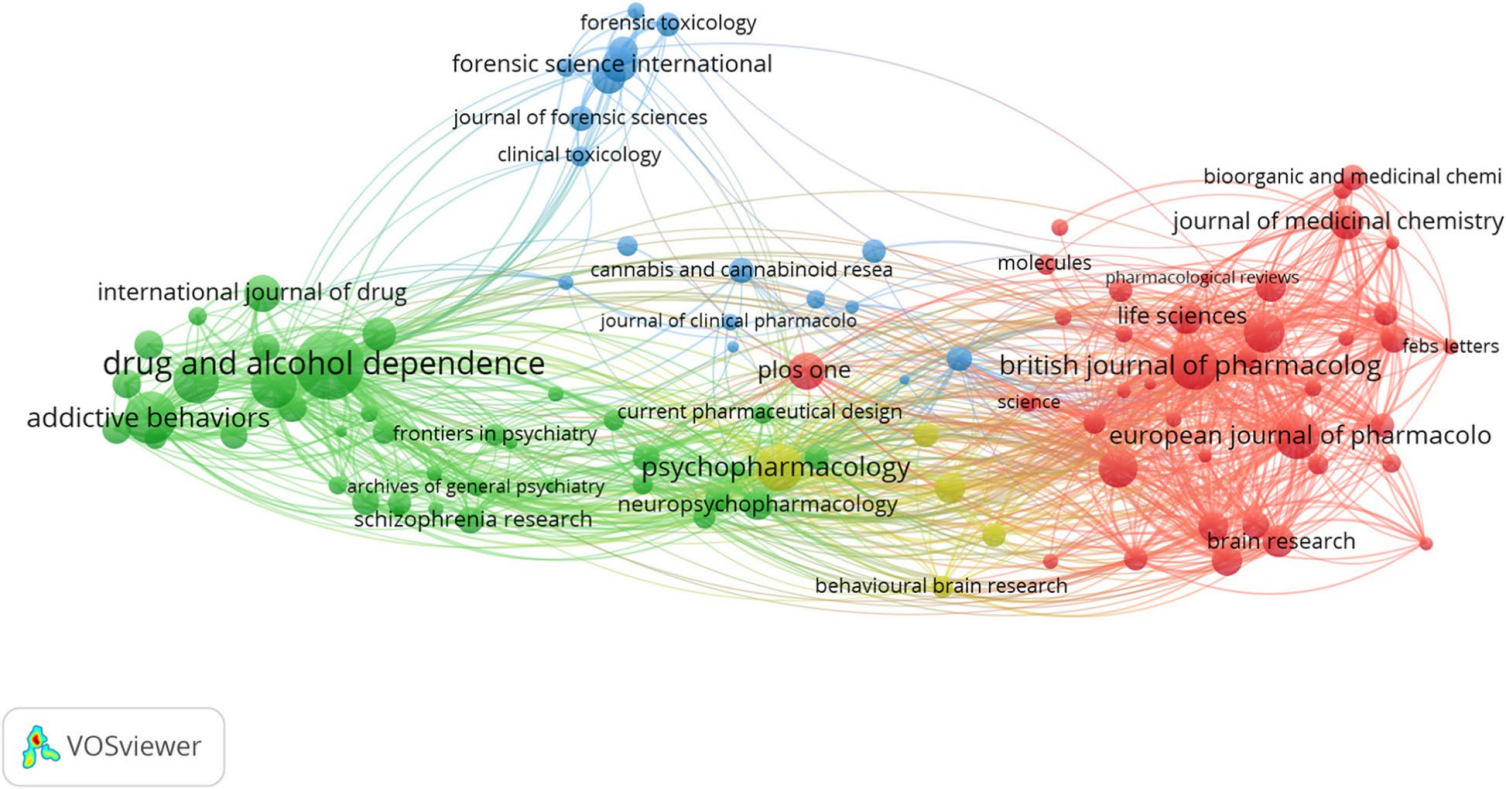
Citation analysis of the 100 journals publishing the largest number of cannabis and cannabinoid publications. Journals with a higher total number of citations on publications are depicted using larger nodes and larger text. The VOSviewer software was used to generate the bibliometric web

A total of 29 802 publications (*n* = 10 214, 34.27% open access), published by 65 109 authors were published in 5474 journals from 1829 to 2021. Since the 1960s, an upward trend with respect to the volume of publication can be observed, with 2020 marking the year with the most publications (Fig. 1 and Supplementary File 1). Drug And Alcohol Dependence published the largest number of publications (*n* = 706), followed by Addictive Behaviors (*n* = 419), and the British Journal of Pharmacology (*n* = 356) (Fig. 2 and Table 1). The 2019 journal impact factor of the 30 highest-publishing journals ranged from 1.214 to 7.730 (Table 1).

**Table 1:** Characteristics of the 30 journals having published the highest number of cannabis and cannabinoid publications

Journal name	Number of publications	2019 impact factor	Country of publication	% of total publications (***n*** = 29 802)
Drug and Alcohol Dependence	706	3.951	Ireland	2.37%
Addictive Behaviors	419	3.645	UK	1.41%
British Journal of Pharmacology	356	7.730	USA	1.20%
Psychopharmacology	343	3.130	Germany	1.15%
Addiction	313	6.343	UK	1.05%
Substance Use and Misuse	308	1.497	UK	1.03%
European Journal of Pharmacology	306	3.263	Netherlands	1.03%
Pharmacology Biochemistry and Behavior	246	2.519	USA	0.83%
Journal of Pharmacology and Experimental Therapeutics	243	3.561	USA	0.82%
Neuropharmacology	222	4.431	UK	0.75%
PLOS One	217	2.740	USA	0.73%
International Journal of Drug Policy	202	4.444	Netherlands	0.68%
Forensic Science International	183	2.108	Ireland	0.61%
Journal of Medicinal Chemistry	181	6.205	USA	0.61%
Journal of Psychoactive Drugs	181	1.859	UK	0.61%
Life Sciences	170	3.647	USA	0.57%
Journal of Analytical Toxicology	166	3.513	USA	0.56%
Brain Research	153	2.733	Netherlands	0.51%
Neuroscience	148	3.056	UK	0.50%
Journal of Neuroscience	142	5.674	USA	0.48%
Neuropsychopharmacology	139	6.751	UK	0.47%
Biochemical Pharmacology	137	4.960	USA	0.46%
Drug Testing and Analysis	133	2.903	UK	0.45%
American Journal of Drug and Alcohol Abuse	129	2.925	USA	0.43%
Journal of Studies on Alcohol and Drugs	123	2.448	USA	0.41%
Psychology of Addictive Behaviors	121	2.780	USA	0.41%
Journal of Drug Issues	120	1.214	USA	0.40%
Journal of Psychopharmacology	118	3.121	UK	0.40%
American Journal on Addictions	113	2.371	USA	0.38%
Drug and Alcohol Review	112	2.472	USA	0.38%

The journal name and number of publications were derived from the single search on Scopus conducted for the bibliometric analysis. The journal impact factor is a ratio of the citations to the number of publications a journal publishes over a given time period. The demographic information of each journal was found using the Scimago Journal & Country Rank

**Fig. 3:**
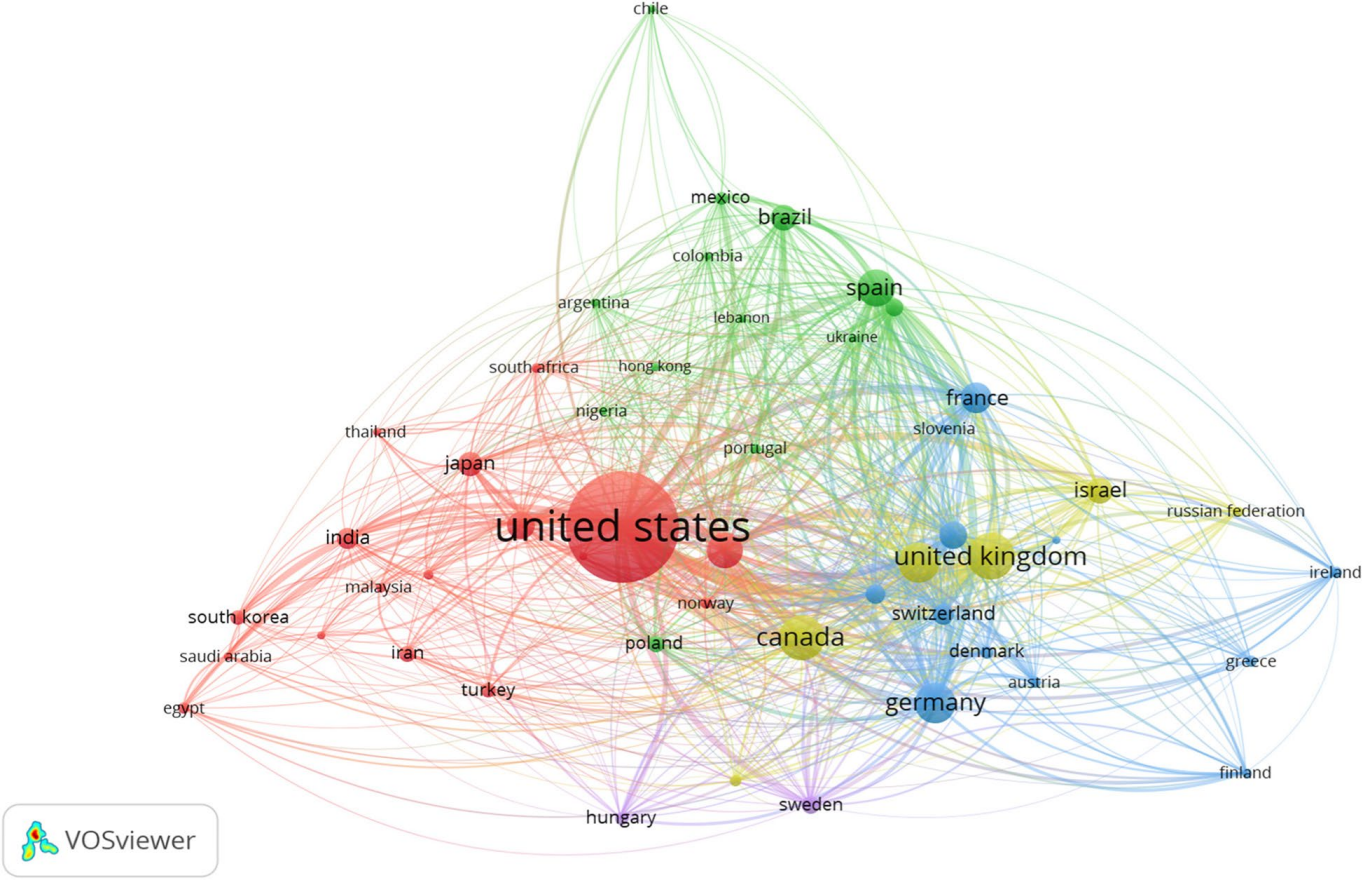
Co-authorship analysis of the 50 most productive countries with respect to cannabis and cannabinoid research. The number of edges connecting two nodes visually quantifies the level of collaboration between researchers or institutions in different countries. Data was collected from Scopus, and the VOSviewer software tool was used to generate the bibliometric web

The subject area containing the largest number of publications was “medicine” (*n* = 17 124, 57.46%), followed by “pharmacology, toxicology and pharmaceutics” (*n* = 8604, 28.87%), then “biochemistry, genetics and molecular biology” (*n* = 5827, 19.55%). Publications were primarily published in English (*n* = 27,715, 93.00%), followed by German (*n* = 658, 2.21%), then French (*n* = 490, 1.64%). The breakdown by document type was article (*n* = 26 296, 83.24%) and review (*n* = 3506, 11.76%). The most common publication countries included the USA (*n* = 12,420, 41.68%), the UK (*n* = 2236, 7.50%), and Canada (*n* = 2062, 6.92%) (Fig. 3). The most common affiliations were the University of Toronto (*n* = 455, 1.53%), King’s College London (*n* = 428, 1.44%), and the National Institutes of Health (*n* = 426, 1.43%); the most common funding sponsors were the National Institutes of Health (*n* = 5848, 19.62%), the US Department of Health and Human Services (*n* = 5778, 19.39%), and the National Institute on Drug Abuse (*n* = 4371, 14.67%) (Table 2). In addition, the 10 most highly-published authors are provided in Table 3, and the 10 most highly-cited publications are provided in Table 4.

**Table 2:** General characteristics of cannabis and cannabinoid publications

Publication volume			
Number of total publications (*n* = 29 802)			
Number of open access publications (*n* = 10 214)		(% of publications)	34.27%
Document type (# of publications)	Article (*n* = 26 296)		88.24%
	Review (*n* = 3506)		11.76%
Source titles (journals) across all publications (*n* = 5474)			
Unique authors across all publications (*n* = 65 109)			
Subject area of publication (10 highest)			
(# of publications)	Medicine (*n* = 17 124)	(% of publications)	57.46%
	Pharmacology, Toxicology and Pharmaceutics (*n* = 8604)		28.87%
	Biochemistry, Genetics and Molecular Biology (*n* = 5827)		19.55%
	Neuroscience (*n* = 4266)		14.31%
	Psychology (*n* = 2542)		8.53%
	Social Sciences (*n* = 2188)		7.34%
	Chemistry (*n* = 2167)		7.27%
	Agricultural and Biological Sciences (*n* = 1578)		5.30%
	Environmental Science (*n* = 864)		2.90%
	Nursing (*n* = 676)		2.27%
Language of publication (10 highest)			
(# of publications)	English (*n* = 27 715)	(% of publications)	93.00%
	German (*n* = 658)		2.21%
	French (*n* = 490)		1.64%
	Spanish (*n* = 362)		1.22%
	Chinese (*n* = 133)		0.45%
	Dutch (*n* = 91)		0.31%
	Italian (*n* = 78)		0.26%
	Portuguese (*n* = 76)		0.26%
	Japanese (*n* = 63)		0.21%
	Norwegian (*n* = 52)		0.17%
Country of publication (10 highest)			
(# of publications)	USA (*n* = 12 420)	(% of publications)	41.68%
	UK (*n* = 2236)		7.50%
	Canada (*n* = 2062)		6.92%
	Germany (*n* = 1694)		5.68%
	Italy (*n* = 1663)		5.58%
	Spain (*n* = 1384)		4.64%
	Australia (*n* = 1325)		4.45%
	France (*n* = 1030)		3.46%
	Netherlands (*n* = 795)		2.67%
	China (*n* = 755)		2.53%
Institutional affiliation (10 highest)			
(# of publications)	University of Toronto (*n* = 455)	(% of publications)	1.53%
	King’s College London (*n* = 428)		1.44%
	National Institutes of Health (*n* = 426)		1.43%
	Virginia Commonwealth University (*n* = 386)		1.30%
	Universidad Complutense de Madrid (*n* = 372)		1.25%
	University of Washington, Seattle (*n* = 354)		1.19%
	National Institute on Drug Abuse (*n* = 348)		1.17%
	Harvard Medical School (*n* = 341)		1.14%
	Inserm (*n* = 335)		1.12%
	Universidade de Sao Paulo (*n* = 328)		1.10%
Funding sponsor (10 highest)			
(# of publications)	National Institutes of Health (USA) (*n* = 5848)	(% of publications)	19.62%
	Department of Health and Human Services (USA) (*n* = 5778)		19.39%
	National Institute on Drug Abuse (USA) (*n* = 4371)		14.67%
	National Institute on Alcohol Abuse and Alcoholism (USA) (*n* = 1007)		3.38%
	National Institute of Mental Health (USA) (*n* = 647)		2.17%
	European Commission (*n* = 434)		1.46%
	Government of Canada (*n* = 394)		1.32%
	National Cancer Institute (USA) (*n* = 317)		1.06%
	Canadian Institutes of Health Research (*n* = 314)		1.05%
	UK Research and Innovation (*n* = 299)		1.00%

VOSviewer was used to find the number of unique authors across all publications and all other data was collected from Scopus. The Department of Health and Human Services, National Institute on Drug Abuse, National Institute on Alcohol Abuse and Alcoholism, National Institute of Mental Health, and National Cancer Institute (all based in the USA) are associated with the US National Institutes of Health. The Canadian Institutes of Health Research is associated with the Government of Canada

**Table 3:** Ten most productive authors across cannabis and cannabinoid publications

Author name	Number of publications	% of total publications (***n*** = 29 802)
Mechoulam, R.	228	0.77%
Makriyannis, A.	225	0.76%
Huestis, M.A.	180	0.60%
Di Marzo, V.	172	0.58%
Martin, B.R.	162	0.54%
Mackie, K.	142	0.48%
Pertwee, R.G.	140	0.47%
Lutz, B.	96	0.32%
Zuardi, A.W.	92	0.31%
Guimarães, F.S.	87	0.29%

All data was collected from Scopus

**Table 4:** Ten highest cited cannabis and cannabinoid publications

Title	Authors	Year	Source title	Cited by
Isolation and structure of a brain constituent that binds to the cannabinoid receptor	Devane W.A., Hanuš L., Breuer A., Pertwee R.G., Stevenson L.A., Griffin G., Gibson D., Mandelbaum A., Etinger A., Mechoulam R.	1992	Science	4340
Structure of a cannabinoid receptor and functional expression of the cloned cDNA	Matsuda L.A., Lolait S.J., Brownstein M.J., Young A.C., Bonner T.I.	1990	Nature	3923
Molecular characterization of a peripheral receptor for cannabinoids	Munro S., Thomas K.L., Abu-Shaar M.	1993	Nature	3788
Identification of an endogenous 2-monoglyceride, present in canine gut, that binds to cannabinoid receptors	Mechoulam R., Ben-Shabat S., Hanus L., Ligumsky M., Kaminski N.E., Schatz A.R., Gopher A., Almog S., Martin B.R., Compton D.R., Pertwee R.G., Griffin G., Bayewitch M., Barg J., Vogel Z.	1995	Biochemical Pharmacology	2166
International Union of Pharmacology. XXVII. Classification of cannabinoid receptors	Howlett A.C., Barth F., Bonner T.I., Cabral G., Casellas P., Devane W.A., Felder C.C., Herkenham M., Mackie K., Martin B.R., Mechoulam R., Pertwee R.G.	2002	Pharmacological Reviews	2092
Determination and characterization of a cannabinoid receptor in rat brain	Devane W.A., Dysarz III F.A., Johnson M.R., Melvin L.S., Howlett A.C.	1988	Molecular Pharmacology	1875
Cannabinoid receptor localization in brain	Herkenham M., Lynn A.B., Little M.D., Johnson M.R., Melvin L.S., De Costa B.R., Rice K.C.	1990	Proceedings of the National Academy of Sciences of the United States of America	1771
Characterization and localization of cannabinoid receptors in rat brain: A quantitative in vitro autoradiographic study	Herkenham M., Lynn A.B., Johnson M.R., Melvin L.S., De Costa B.R., Rice K.C.	1991	Journal of Neuroscience	1674
2-arachidonoylglycerol: A possible endogenous cannabinoid receptor ligand in brain	Sugiura T., Kondo S., Sukagawa A., Nakane S., Shinoda A., Itoh K., Yamashita A., Waku K.	1995	Biochemical and Biophysical Research Communications	1670
Isolation, structure, and partial synthesis of an active constituent of hashish	Gaoni Y., Mechoulam R.	1964	Journal of the American Chemical Society	1583

All data was collected from Scopus

### Landscape of cannabis and cannabinoid research over decades

Since the 1980s, an increase in the volume of open access publications was observed, with the 2010s marking the decade with the highest percentage of open access versus subscription publications (*n* = 6745, 48.92%). Between the 1960s and 2010s, a steady increase in the number of publications published in the areas of “immunology and microbiology,” “neuroscience,” “nursing,” “psychology,” and “social sciences” was observed. The subject area that consistently contributed to the highest proportion of cannabis publications was “medicine,” with the 2010s marking the decade with the highest percentage of all cannabis publications (*n* = 8460, 61.36%). The “biochemistry, genetics and molecular biology” and “pharmacology, toxicology, and pharmaceutics” reflect the subject areas with the second and third highest proportion of publications across the six decades. The number of publications pertaining to the former has ranged from 16.27 to 30.81%, while the latter between 20.93 and 34.67% of the published literature, across these six decades.

Multiple journals have consistently ranked as a top ten publisher of cannabis and cannabinoid literature across all multiple decades. Examples include the journals “Drug and Alcohol Dependence” (top ten from 1980 to 2019), “Psychopharmacology” (top ten from 1980 to 2019), and the “British Journal of Pharmacology” (top ten from 1990 to 2019). With respect to regional productivity, the USA has consistently generated the highest percentage of publications across each decade, with a notable increase between the decades of 1970-1979 (*n* = 979, 41.68%) and 1960–1969 (*n* = 33, 10.96%). This is supported by the shift in the top 10 funding sponsors of research beginning in the 1970s, where it is observed that an increasing number of publications have been supported by US funders; this includes the National Institutes of Health, the US Department of Human and Health Services, and the National Institute of Drug Abuse from 1970 to 1979 (*n* = 45, 1.92%; *n* = 46, 1.96%; *n* = 25, 1.06% respectively) to 1980–1989 (*n* = 208, 13.38%; *n* = 200, 12.86%; *n* = 167, 10.74%; respectively). In more recent years, additional US funders have supported cannabis and cannabinoid research, including the National Institute of Mental Health and the National Institute of Alcohol Abuse and Alcoholism.

Another country that has consistently remained one of the top ten most productive countries includes the UK, which rose to the top three from the 1970s onwards. This rise to the top three coincides with the Presbyterian Historical Society and UK Research and Innovation, both UK institutions, as tied for the highest-ranking non-US funding sponsors in the 1970s. Similarly, Germany, another country that has remained in the top ten highest producing countries, entered the top three in the 2000s for the first time with Deutsche Forschungsgemeinschaft as the highest-ranking non-US funding sponsor. This is also observed in Canada, which re-entered the top three in 2010s with the Canadian Institutes of Health as the highest-ranking non-US funding sponsor. Despite the top ten funding sponsors across each decade being occupied by institutions from the top producing countries, the top ten affiliations of authors are from a more diverse group of countries. For example, the top author affiliation in the 2000s was the Universidad Complutense de Madrid, an institution in Spain, which was the fifth-highest producing country of the decade. Similarly, the Universidade de São Paulo was ranked fourth in the 2010s, while Brazil was not in the top ten highest producing countries. Lastly, with respect to language, most publications have been written in English across all decades, with the 2010s marking the greatest disparity between publications in English (*n* = 13 078, 94.85%); other most common languages of publication included German, Spanish, and French, albeit proportionately far less than English.

Raw cannabis and cannabinoid research bibliometric data collected on a decade-by-decade basis between the 1960s and the 2010s, including and beyond that described here, are found in Supplementary File 1.

### Intersecting topics

**Fig. 4:**
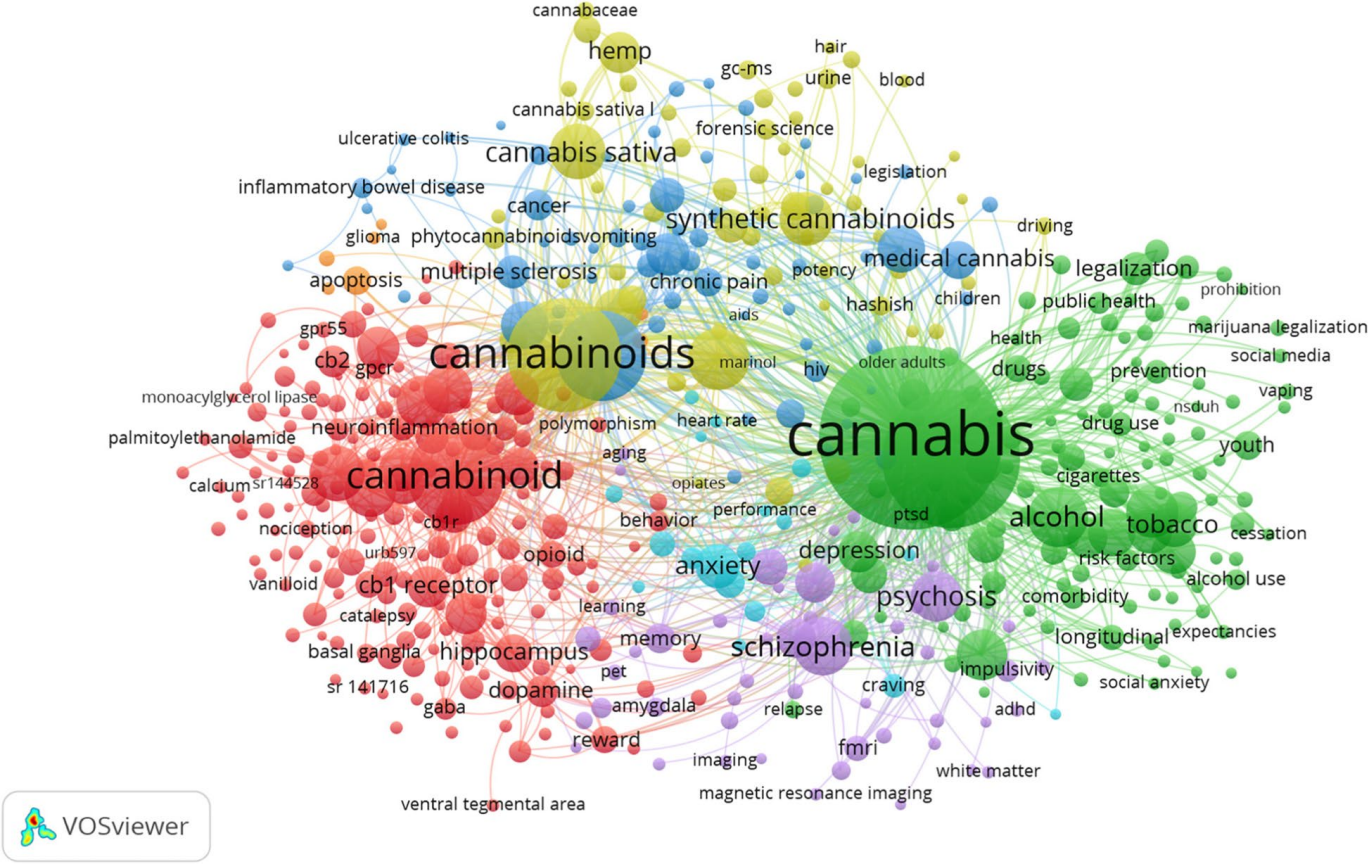
Co-occurrence analysis of the 500 most frequent author keywords. The most frequently used keywords appear as larger nodes with larger text. Each cluster of highly related keywords is displayed using a different colour to facilitate categorization. Data was collected from Scopus, and the bibliometric web was generated using the VOSviewer software

Cannabis and cannabinoid publications were found to intersect with a wide variety of topics, including mental health disorders, disease, and the usage of other substances. Within the cluster of mental health-related topics, it was found that many publications included the keywords “psychosis” (*n* = 395), “depression” (*n* = 256), “schizophrenia” (*n* = 529), and “anxiety” (*n* = 313) (Table 5). Within the realm of disease, publications were written at the intersection of cannabis and chronic diseases, such as multiple sclerosis (*n* = 178) and cancer (*n* = 144). Additionally, it was found publications were also written at the intersection of the usage of cannabis/cannabinoids and that of other substances, as shown by the cluster of substance-related keywords (Fig. 4) including “alcohol” (*n* = 571) and “tobacco” (*n* = 327).

**Table 5:** Sixty most frequent author keywords across the titles of cannabis and cannabinoid publications

Author keyword	Frequency	%
Cannabis	5171	17.35%
Marijuana	2876	9.65%
Cannabinoids	1998	6.70%
Cannabinoid	1417	4.75%
Cannabidiol	1237	4.15%
THC	604	2.03%
Alcohol	571	1.92%
Schizophrenia	529	1.78%
Cannabis Sativa	486	1.63%
Anandamide	467	1.57%
Synthetic Cannabinoids	448	1.50%
Adolescents	436	1.46%
Cannabinoid Receptors	434	1.46%
Cannabinoid Receptor	419	1.41%
Adolescence	409	1.37%
Psychosis	395	1.33%
Endocannabinoids	383	1.29%
Medical Marijuana	356	1.19%
Tetrahydrocannabinol	350	1.17%
Cannabis Use	348	1.17%
Pain	343	1.15%
Tobacco	327	1.10%
Anxiety	313	1.05%
Endocannabinoid System	311	1.04%
Endocannabinoid	307	1.03%
Substance Use	304	1.02%
Marijuana Use	299	1.00%
Inflammation	292	0.98%
CBD	280	0.94%
CB1 Receptor	275	0.92%
Hemp	275	0.92%
CB1	272	0.91%
Rat	268	0.90%
Depression	256	0.86%
Adolescent	248	0.83%
Cognition	244	0.82%
Addiction	242	0.81%
Medical Cannabis	236	0.79%
Hippocampus	226	0.76%
Δ9-Tetrahydrocannabinol	223	0.75%
Dependence	214	0.72%
Rimonabant	212	0.71%
Cannabis Use Disorder	211	0.71%
Cocaine	207	0.69%
Epilepsy	191	0.64%
Treatment	191	0.64%
Synthetic Cannabinoid	179	0.60%
Epidemiology	178	0.60%
Multiple Sclerosis	178	0.60%
Legalization	177	0.59%
Neuroprotection	170	0.57%
Dopamine	163	0.55%
Young Adults	160	0.54%
CB2	158	0.53%
Obesity	158	0.53%
Pregnancy	156	0.52%
Memory	150	0.50%
Smoking	149	0.50%
Cancer	144	0.48%
Withdrawal	139	0.47%

The most common author keywords for this subset of publications (*n* = 29 802). All data was downloaded from Scopus and analyzed in VOSviewer

## Discussion

The objective of the present bibliometric analysis is to capture the characteristics of scholarly journal publications on the topic of cannabis research. The search conducted on Scopus yielded nearly 30 000 publications, representing the largest bibliometric analysis of cannabis literature to date to the authors’ knowledge. An increase in the volume of literature was observed beginning in the 1960s; however, the most recent 20 years have represented the largest increase in the volume of research published on the topic of cannabis. This can largely be attributed to the fact that more than $1.5 billion in funding has been allocated to cannabis research just between 2000 and 2018 (Hellth n.d.-a). One analysis provided a breakdown of this collective funding by country, finding that more than $1.4 billion funded researchers in the USA; the UK at $39.9 million, and Canada at $36.1 million represented a distant second and third respectively (Hellth n.d.-a). In the present study, it was found that the USA was by far the most productive country with respect to cannabis research (*n* = 12 420) publications, followed by the UK (*n* = 2236) and Canada (*n* = 2062), matching the order and approximate proportions as found by the aforementioned analysis. The data also suggests that the presence of funding may contribute to an increase in the productivity of a country, as shown by the first-time appearance of Germany and Canada in the top 3 most productive countries in decades where German and Canadian institutions were included in the top 10 funding sponsors. It is also unsurprising that out of the top 10 institutional affiliations responsible for publishing this cannabis research, five originate from the USA, one from the UK, and one from Canada. Additionally, with respect to funding sponsors, six are based in the USA, two in Canada, and one in the UK (excluding the European Commission). It has also been found that more cannabis research has focused on the harms associated with the substance, as opposed to its medical uses, especially in the USA (Science n.d.; Hellth n.d.-b). This is also reflected in the present study’s findings, as nearly half (*n* = 14) of the 30 journals have published the highest number of cannabis publications containing harm-associated words in their titles such as “dependence,” “addictive/addiction,” “forensic,” “drug,” and “abuse.” In contrast, only a single journal—the Journal of Medicinal Chemistry—exclusively publishes medical cannabis research, while the remaining journals have titles indicating that they publish a mixture of cannabis research relating both to harms and medical properties (e.g., pharmacology journals).

### Intersectionality with mental health conditions

Cannabis is a topic of interest when investigating the onset and treatment of mental health conditions, as indicated by the prevalence of the author keywords “psychosis” (*n* = 395), “schizophrenia” (*n* = 529), and “anxiety” (*n* = 313) as shown in Fig. 4. Prior systematic reviews, such as Crippa et al. (2009) and Walsh et al. (2017) analyzed cannabis as an anxiolytic and its use among patients suffering from anxiety. They found that recent studies supported (Walsh et al. 2017) the application of cannabis as an anxiolytic (Bonn-Miller et al. 2014; Grella et al. 2014; Walsh et al. 2013; Webb and Webb 2014) for patients living with anxiety and suggested a need for further research to clarify the mechanism by which cannabis relieves anxiety (Crippa et al. 2009). Although a few studies, such as that by Kedzior and Laeber (2014) suggested a positive correlation between anxiety and cannabis use, others suggested this correlation may be caused by other underlying associations with factors that influence anxiety susceptibility or the non-medical use of cannabis (Walsh et al. 2017; Johnson et al. 2010; Zvolensky et al. 2009).

Systematic reviews and meta-analyses have generally found a consistent association between cannabis use and psychosis or other mental health conditions that cause psychotic symptoms (Matheson et al. 2011; Minozzi et al. 2010; Moore et al. 2007). Johnson et al. (2010) and Minozzi et al. (2010) argued that a further need for research into the longitudinal effects of cannabis use with respect to psychosis. Malone et al. (2010) and Shapiro et al. (2010) have investigated the long-term effects of cannabis use on brain development and susceptibility to psychosis (Malone et al. 2010; Shapiro and Buckley-Hunter 2010). Meta-analyses also suggest an earlier onset of psychotic symptoms in individuals who used cannabis compared to non-users (Large et al. 2011), which is further supported by findings indicating an increased susceptibility in individuals with a genetic vulnerability to psychosis (McLaren et al. 2010; Proal et al. 2014).

### Intersectionality with chronic illness

Considerable research has explored the potential of cannabis as a treatment for managing pain associated with chronic conditions; in Fig. 4, we can see that author keywords for a number of chronic conditions are depicted by large nodes, including multiple sclerosis (*n* = 178), cancer (*n* = 144), and pain (*n* = 343). One systematic review conducted by Belendiuk et al. (2015) provides a review of the literature exploring the use of cannabis as a treatment for cancer and multiple sclerosis. It was found that there were conflicting results associated with the efficacy of cannabinoids when used to relieve symptoms of cancer treatment (Belendiuk et al. 2015). Other studies have described a reduction in local tumor growth in patients prescribed drugs derived from cannabinoids (Abrams and Guzman 2015; Bowles et al. 2012), whereas others report that tumor growth is increased in patients due to the suppression of immune system components (McKallip et al. 2005). Such contradictions in the available evidence may be a reason why others have further suggested that there is insufficient evidence to accurately describe the potential of cannabis and cannabinoids as treatments for cancer (Belendiuk et al. 2015).

Belendiuk et al. (2015) also explored the use of cannabis as a treatment for multiple sclerosis. Studies have reported that cannabis administered orally or sublingually may relieve common symptoms of MS, such as muscle spasticity (Lakhan and Rowland 2009; Novotna et al. 2011; Wade et al. 2010) and lead to improvement in the long term (Notcutt et al. 2012; Serpell et al. 2013). However, there is also evidence of a decrease in cognitive function in patients with multiple sclerosis who use cannabis compared to their non-using counterparts (Corey-Bloom et al. 2012; Honarmand et al. 2011; Pavisian et al. 2014). Such inconsistencies may be associated with the reliance on self-reporting as a component of the study methodology and that more studies are required to evaluate the potential of cannabis as a treatment for multiple sclerosis (Belendiuk et al. 2015). Nugent et al. (2017) conducted a systematic review to investigate current research into the application of cannabis as a treatment for chronic pain. It was found that although many studies suggest the efficacy of cannabis as a treatment for neuropathic pain (Lynch et al. 2014; Serpell et al. 2014; Wallace et al. 2015; Wilsey et al. 2013; Wilsey et al. 2016), there is insufficient evidence to definitively suggest its efficacy as a treatment for other chronic pain conditions, such as arthritis and fibromyalgia (Nugent et al. 2017).

### Intersectionality with other substances

There is a significant volume of research investigating the intersectionality of cannabis and other substances, particularly alcohol (*n* = 571) and tobacco (*n* = 327), as evidenced by the author keywords presented in Fig. 4. Several systematic reviews have explored the relationship between cannabis use and other substances, such as tobacco and alcohol (Karoly et al. 2020; Peters et al. 2012). There is evidence suggesting an association between tobacco and cannabis co-use and a greater likelihood of both lifetime and current cannabis use disorders (Agrawal et al. 2009; Agrawal and Lynskey 2009; Timberlake 2009), as well as greater symptoms of withdrawal when ceasing to use cannabis (Vandrey et al. 2008). To further understand the interaction between alcohol and cannabis use, it was recommended that future studies incorporate methods to accurately assess the frequency of cannabis use (Karoly et al. 2020).

With respect to alcohol, some research has suggested that co-use is associated with an increase in the frequency of consumption of both substances (Hayaki et al. 2016; Norberg et al. 2012; Patrick et al. 2018) and increased psychological distress (Kelly et al. 2015a). Researchers have drawn mixed conclusions on the effect of alcohol and cannabis co-use on neurological development. Some evidence has been found that co-use may result in more positive physiological effects, compared to alcohol consumption alone (Ewing et al. 2014; Infante et al. 2018), whereas others have reported further negative effects, such as poor academic performance (Kelly et al. 2015b). As such, further large-scale longitudinal studies are needed for a more comprehensive understanding of how cannabis and its intersection with alcohol impact neurological development (Karoly et al. 2020; Brown et al. 2015; Scholes-Balog et al. 2016). The large sample size of this experimental design would allow for a more thorough assessment of the intersection of cannabis and other environmental factors (Karoly et al. 2020).

### Strengths and limitations

This bibliometric study contained several notable strengths including the fact that the characteristics of 29 802 publications published in 5474 journals were captured, representing the largest of all bibliometric analyses on this topic to date. Searches were conducted on Scopus as this academic database has a larger coverage in comparison to other databases such as Web of Science; this decision was also justified by the fact that one study conducted cannabis-specific searches on Scopus in addition to other databases, finding that no search results were lost when compared to searching only the former (Matielo et al. 2018). Despite this, it is always possible that some literature may not have been captured by not searching other databases, however, this would have introduced considerable complexities with respect to the ability to analyze search results efficiently (e.g., deduplication of such a large volume of publications). One additional limitation includes the fact that search results were not screened manually, even though this is not typically done for bibliometric analyses that include more than a couple of thousand publications, as such a step is neither practical nor efficient. This was mitigated by the search strategy selected; fortunately, publications on the topic of cannabis and cannabinoid almost always include one of the following search strings inclusive of cannabi*, hashish, marijuana, or marihuana in their title, in combination with the fact that these same search strings very infrequently refer to a non-cannabis/cannabinoid topic. Despite the existence of many slang terms used to refer to cannabis, it is extremely uncommon for authors to use such a term in the title of their article. Lastly, the methodology is further limited by the search being restricted to the title of the published research. This restriction prevents the recognition of recurring trends in other areas, such as recurring methodology and common descriptors included in the publication abstract, though given the topic area it can be assumed that the vast majority of the published literature with a primary focus on cannabis would include this term (or an appropriate synonym we captured) in the article title.

## Conclusions

The present study captured the characteristics of scholarly journal publications on the topic of cannabis and cannabinoid research literature, yielding nearly 30 000 publications, representing the largest bibliometric analysis conducted on this topic to date. The most productive countries included the USA, the UK, and Canada; unsurprisingly, a large proportion of institutional affiliations and funding sponsors associated with this subset of publications also originated from these three countries. Over the past 20 years, the volume of cannabis research has grown steeply, which can be largely attributed to the existence of a large amount of funding recently dedicated to research on this topic.

## Supplementary Information

**Additional file 1.** Decade-by-Decade Cannabis and Cannabinoid Research Bibliographic Data (1960s-2010s).

## Data Availability

All data generated or analyzed during this study are included in this published article.

## Abbreviations

CBD: Cannabidiol
THC: Tetrahydrocannabinol
