# Supplementary material for: A bibliometric analysis of the cannabis and cannabinoid research literature
Source: J Cannabis Res. 2022 May 25;4:25. doi: 10.1186/s42238-022-00133-0 (PMC9131698; doi:10.1186/s42238-022-00133-0)
Supplement: Supplementary file 1 — Additional file 1. Decade-by-Decade Cannabis and Cannabinoid Research Bibliographic Data (1960s-2010s). [file 42238_2022_133_MOESM1_ESM.pdf]

Supplementary File 1: Decade-by-Decade Cannabis and Cannabinoid Research Bibliographic Data (1960s-2010s)

Decade-by-Decade Breakdown by Open Access Status (1960s-2010s)

Decade	Open Access	% of Total	Subscription	% of Total
1960-1969 (n=301)	28	9.30	273	90.70
1970-1979 (n=2349)	86	3.66	2263	96.34
1980-1989 (n=1555)	49	3.15	1506	96.85
1990-1999 (n=2071)	252	12.17	1819	87.83
2000-2009 (n=5995)	1602	26.72	4393	73.28
2010-2019 (n=13788)	6745	48.92	7043	51.08

Decade-by-Decade Breakdown by Subject Area (1960s-2010s)

Subject Area	Decade											
	1960-1969		1970-1979		1980-1989		1990-1999		2000-2009		2010-2019	
	# of Publications	% of Total	# of Publications	% of Total	# of Publications	% of Total	# of Publications	% of Total	# of Publications	% of Total	# of Publications	% of Total
Agricultural and Biological Sciences	25	8.31	79	3.36	54	3.47	115	5.55	273	4.55	668	4.84
Arts and Humanities	5	1.66	116	4.94	15	0.96	25	1.21	73	1.22	137	0.99
Biochemistry, Genetics and Molecular Biology	58	19.27	473	20.14	341	21.93	638	30.81	1385	23.10	2243	16.27
Business, Management and Accounting	0	0.00	5	0.21	4	0.26	2	0.10	7	0.12	83	0.60
Chemical Engineering	15	4.98	23	0.98	40	2.57	54	2.61	74	1.23	257	1.86
Chemistry	54	17.94	152	6.47	157	10.10	139	6.71	311	5.19	968	7.02
Computer Science	0	0.00	4	0.17	0	0.00	4	0.19	6	0.10	65	0.47
Decision Sciences	0	0.00	0	0.00	0	0.00	1	0.05	5	0.08	8	0.06
Dentistry	0	0.00	0	0.00	1	0.06	4	0.19	6	0.10	31	0.22
Earth and Planetary Sciences	2	0.66	12	0.51	2	0.13	7	0.34	3	0.05	21	0.15
Economics, Econometrics and Finance	0	0.00	3	0.13	1	0.06	3	0.14	21	0.35	90	0.65
Energy	0	0.00	0	0.00	0	0.00	3	0.14	7	0.12	40	0.29
Engineering	0	0.00	7	0.30	4	0.26	9	0.43	30	0.50	142	1.03
Environmental Science	3	1.00	29	1.23	68	4.37	64	3.09	87	1.45	456	3.31
Health Professions	0	0.00	5	0.21	10	0.64	6	0.29	22	0.37	105	0.76
Immunology and Microbiology	4	1.33	12	0.51	16	1.03	32	1.55	113	1.88	289	2.10
Materials Science	7	2.33	0	0.00	4	0.26	11	0.53	27	0.45	108	0.78
Mathematics	0	0.00	1	0.04	3	0.19	2	0.10	4	0.07	27	0.20
Medicine	159	52.82	1335	56.83	877	56.40	894	43.17	3122	52.08	8460	61.36
Multidisciplinary	15	4.98	58	2.47	6	0.39	34	1.64	49	0.82	321	2.33
Neuroscience	4	1.33	147	6.26	102	6.56	351	16.95	1176	19.62	2023	14.67
Nursing	1	0.33	14	0.60	18	1.16	18	0.87	108	1.80	362	2.63
Pharmacology, Toxicology and Pharmaceutics	63	20.93	641	27.29	502	32.28	718	34.67	1971	32.88	3902	28.30

Physics and Astronomy	0	0.00	6	0.26	2	0.13	5	0.24	11	0.18	61	0.44
Psychology	18	5.98	131	5.58	142	9.13	124	5.99	468	7.81	1340	9.72
Social Sciences	11	3.65	140	5.96	147	9.45	133	6.42	331	5.52	1126	8.17
Veterinary	0	0.00	10	0.43	8	0.51	3	0.14	12	0.20	39	0.28
Undefined	0	0.00	1	0.04	3	0.19	3	0.14	0	0.00	1	0.01

Decade-by-Decade Breakdown by Top 10 Source Titles (1960s-2010s)

1960-1969			1970-1979			1980-1989		
**Source Title**	**# of Publications**	**% of Total**	**Source Title**	**# of Publications**	**% of Total**	**Source Title**	**# of Publications**	**% of Total**
Journal of Psychoactive Drugs	11	3.65	Federation Proceedings	53	2.26	Substance Use and Misuse	36	2.32
Lancet	11	3.65	Psychopharmacologia	52	2.21	Journal of Psychoactive Drugs	34	2.19
Tetrahedron Letters	9	2.99	Annals of the New York Academy of Sciences	51	2.17	Pharmacology Biochemistry and Behavior	33	2.12
American Journal of Psychiatry	8	2.66	Substance Use and Misuse	48	2.04	Psychopharmacology	32	2.06
Journal of the American Chemical Society	8	2.66	American Journal of Psychiatry	38	1.62	NIDA Research Monograph Series	30	1.93
Science	8	2.66	Journal of Chromatography A	38	1.62	NIDA Research Monograph	28	1.80
Archives Internationales de Pharmacodynamie et de Therapie	7	2.33	Advances in the Biosciences	35	1.49	Bulletin on Narcotics	27	1.74
Bulletin of the National Institute of Hygienic Sciences	7	2.33	British Journal of Addiction to Alcohol Other Drugs	34	1.45	Journal of Analytical Toxicology	27	1.74
Tetrahedron	7	2.33	Bulletin on Narcotics	34	1.45	Drug and Alcohol Dependence	26	1.67
Fresenius Zeitschrift Fur Analytische Chemie (Tied for Top 10)	6	1.99	Journal of Pharmacy and Pharmacology	34	1.45	Federation Proceedings	23	1.48
JAMA the Journal of The American Medical Association (Tied for Top 10)	6	1.99						
Psychopharmacologia (Tied for Top 10)	6	1.99						

1990-1999			2000-2009			2010-2019		
**Source Title**	**# of Publications**	**% of Total**	**Source Title**	**# of Publications**	**% of Total**	**Source Title**	**# of Publications**	**% of Total**
Life Sciences	65	3.14	European Journal of Pharmacology	162	2.70	Drug and Alcohol Dependence	437	3.17
Pharmacology Biochemistry and Behavior	64	3.09	British Journal of Pharmacology	145	2.42	Addictive Behaviors	261	1.89
Journal of Pharmacology and Experimental Therapeutics	58	2.80	Drug and Alcohol Dependence	116	1.93	Plos One	185	1.34

European Journal of Pharmacology	53	2.56	Addiction	95	1.58	Addiction	151	1.10
Journal of Analytical Toxicology	37	1.79	Psychopharmacology	91	1.52	British Journal of Pharmacology	147	1.07
Brain Research	31	1.50	Journal of Pharmacology and Experimental Therapeutics	90	1.50	International Journal of Drug Policy	137	0.99
British Journal of Pharmacology	29	1.40	Journal of Neuroscience	81	1.35	Psychopharmacology	137	0.99
Drug and Alcohol Dependence	28	1.35	Neuropharmacology	80	1.33	Substance Use and Misuse	121	0.88
Substance Use and Misuse	28	1.35	Addictive Behaviors	78	1.30	Forensic Science International	118	0.86
Journal of Medicinal Chemistry (Tied for Top 10)	25	1.21	Neuroscience	66	1.10	Drug Testing and Analysis	111	0.81
Psychopharmacology (Tied for Top 10)	25	1.21						

Decade-by-Decade Breakdown by Top 10 Author Keywords (1960s-2010s)

**1960-1969**			**1970-1979**			**1980-1989**		
**Author Keyword**	**# of Publications**	**% of Total**	**Author Keyword**	**# of Publications**	**% of Total**	**Author Keyword**	**# of Publications**	**% of Total**
Article	202	67.11	Cannabis	1821	77.52	Cannabis	968	62.25
Cannabis	194	64.45	Human	907	38.61	Human	775	49.84
Human	111	36.88	Article	862	36.70	Tetrahydrocannabinol	510	32.80
Addiction	69	22.92	Tetrahydrocannabinol	813	34.61	Male	459	29.52
Substance-Related Disorders	69	22.92	Male	693	29.50	Central Nervous System	413	26.56
Adult	45	14.95	Theoretical Study	689	29.33	Cannabinoids	384	24.69
Male	43	14.29	Animal	438	18.65	Female	318	20.45
Female	32	10.63	Adult	419	17.84	Nonhuman	301	19.36
Humans	32	10.63	Substance-Related Disorders	410	17.45	Adult	298	19.16
Drug Effect	31	10.30	Drug Effect	332	14.13	Animal Experiment (Tied for Top 10)	297	19.10
						Cannabinoid (Tied for Top 10)	297	19.10

**1990-1999**			**2000-2009**			**2010-2019**		
**Author Keyword**	**# of Publications**	**% of Total**	**Author Keyword**	**# of Publications**	**% of Total**	**Author Keyword**	**# of Publications**	**% of Total**
Article	1511	72.96	Article	4436	73.99	Article	8668	62.87
Priority Journal	949	45.82	Human	3164	52.78	Human	8551	62.02
Human	935	45.15	Priority Journal	2906	48.47	Humans	7319	53.08
Cannabis	875	42.25	Controlled Study	2827	47.16	Male	6586	47.77
Male	843	40.70	Male	2795	46.62	Cannabis	5767	41.83
Nonhuman	784	37.86	Cannabis	2561	42.72	Priority Journal	5062	36.71
Controlled Study	663	32.01	Humans	2492	41.57	Female	4975	36.08
Cannabinoid	595	28.73	Nonhuman	2460	41.03	Controlled Study	4918	35.67
Cannabinoid Receptor	583	28.15	Animals	1973	32.91	Adult	4445	32.24
Cannabinoids	532	25.69	Female	1696	28.29	Nonhuman	3675	26.65

Decade-by-Decade Breakdown by Top 10 Institutional Affiliations (1960s-2010s)

1960-1969			1970-1979			1980-1989		
**Affiliation**	**# of Publications**	**% of Total**	**Affiliation**	**# of Publications**	**% of Total**	**Affiliation**	**# of Publications**	**% of Total**
Weizmann Institute of Science Israel	9	2.99	VA Medical Center	65	2.77	Centre for Addiction and Mental Health	28	1.80
Universität Bonn	8	2.66	University of Mississippi	48	2.04	University of Mississippi	26	1.67
Faculty of Medicine	6	1.99	Medical College of Virginia	38	1.62	National Institute on Drug Abuse NIDA	21	1.35
Kyushu University	5	1.66	University of California, Los Angeles	35	1.49	Johns Hopkins School of Medicine	19	1.22
Hokkaido University	5	1.66	Universidade Federal de São Paulo	34	1.45	Hebrew University of Jerusalem	19	1.22
USDA Agricultural Research Service (Tied for Top 10)	3	1.00	Eli Lilly and Company	32	1.36	David Geffen School of Medicine at UCLA	18	1.16
Hebrew University of Jerusalem (Tied for Top 10)	3	1.00	University of Mississippi, Research Institute Pharmaceutical Science	28	1.19	University of Mississippi, Research Institute Pharmaceutical Science	18	1.16
University of Toronto (Tied for Top 10)	3	1.00	Harvard Medical School	25	1.06	Hokuriku University	17	1.09
Instituto Biologico - Sao Paulo (Tied for Top 10)	3	1.00	University of Utah School of Medicine	24	1.02	Faculty of Medicine	17	1.09
United States Department of Agriculture (Tied for Top 10)	3	1.00	Virginia Commonwealth University (Tied for Top 10)	23	0.98	University of California, Los Angeles	17	1.09
Arthur D Little, Inc. (Tied for Top 10)	3	1.00	Indiana University School of Medicine (Tied for Top 10)	23	0.98			

1990-1999			2000-2009			2010-2019		
**Affiliation**	**# of Publications**	**% of Total**	**Affiliation**	**# of Publications**	**% of Total**	**Affiliation**	**# of Publications**	**% of Total**
Medical College of Virginia	95	4.59	Universidad Complutense de Madrid	139	2.32	King's College London	284	2.06
Virginia Commonwealth University	88	4.25	National Institutes of Health NIH	120	2.00	University of Toronto	264	1.91
Sanofi S.A.	52	2.51	University of Washington	109	1.82	National Institutes of Health NIH	263	1.91
University of Connecticut	51	2.46	Virginia Commonwealth University	108	1.80	Universidade de São Paulo	221	1.60

Universidad Complutense de Madrid	48	2.32	Consiglio Nazionale delle Ricerche	108	1.80	National Institute on Drug Abuse NIDA	207	1.50
Hebrew University of Jerusalem	44	2.12	University of Aberdeen	91	1.52	UNSW Sydney	189	1.37
National Institute of Mental Health NIMH	41	1.98	National Institute on Drug Abuse NIDA	91	1.52	Harvard Medical School	183	1.33
Faculty of Medicine	38	1.83	Inserm	90	1.50	Inserm	177	1.28
University of Aberdeen	36	1.74	King's College London	73	1.22	Columbia University	170	1.23
Brown University	33	1.59	University of Connecticut	71	1.18	Consiglio Nazionale delle Ricerche	167	1.21

Decade-by-Decade Breakdown by Top 10 Funding Sponsors (1960s-2010s)

**1960-1969**			**1970-1979**			**1980-1989**		
**Funding Sponsor**	**# of Publications**	**% of Total**	**Funding Sponsor**	**# of Publications**	**% of Total**	**Funding Sponsor**	**# of Publications**	**% of Total**
National Institutes of Health	3	1.00	U.S. Department of Health and Human Services	46	1.96	National Institutes of Health	208	13.38
U.S. Department of Health and Human Services	3	1.00	National Institutes of Health	45	1.92	U.S. Department of Health and Human Services	200	12.86
U.S. Public Health Service	3	1.00	National Institute on Drug Abuse	25	1.06	National Institute on Drug Abuse	167	10.74
National Institute of Mental Health	2	0.66	U.S. Public Health Service	22	0.94	National Institute of Neurological Disorders and Stroke	20	1.29
Boston University (Tied for Top 10)	1	0.33	National Institute of Mental Health	13	0.55	National Eye Institute	14	0.90
Business Finland (Tied for Top 10)	1	0.33	Medical Research Council	4	0.17	Eunice Kennedy Shriver National Institute of Child Health and Human Development	13	0.84
Fundação de Amparo à Pesquisa do Estado de São Paulo (Tied for Top 10)	1	0.33	National Eye Institute	4	0.17	National Institute of Mental Health	11	0.71
Medical Research Council (Tied for Top 10)	1	0.33	Fundação de Amparo à Pesquisa do Estado de São Paulo (Tied for Top 10)	3	0.13	U.S. Public Health Service	11	0.71
Medicinska Forskningsrådet (Tied for Top 10)	1	0.33	Presbyterian Historical Society (Tied for Top 10)	3	0.13	National Institute on Alcohol Abuse and Alcoholism	10	0.64
U.S. Food and Drug Administration (Tied for Top 10)	1	0.33	U.S. Department of Justice (Tied for Top 10)	3	0.13	National Center for Research Resources	9	0.58
UK Research and Innovation (Tied for Top 10)	1	0.33	UK Research and Innovation (Tied for Top 10)	3	0.13			
			University of California, Los Angeles (Tied for Top 10)	3	0.13			

1990-1999			2000-2009			2010-2019		
**Funding Sponsor**	**# of Publications**	**% of Total**	**Funding Sponsor**	**# of Publications**	**% of Total**	**Funding Sponsor**	**# of Publications**	**% of Total**
National Institute on Drug Abuse	505	24.38	National Institute on Drug Abuse	1122	18.72	National Institute on Drug Abuse	2249	16.31
National Institutes of Health	404	19.51	National Institutes of Health	812	13.54	National Institutes of Health	1353	9.81
U.S. Department of Health and Human Services	360	17.38	U.S. Department of Health and Human Services	610	10.18	National Institute on Alcohol Abuse and Alcoholism	683	4.95
National Institute of Neurological Disorders and Stroke	45	2.17	National Institute on Alcohol Abuse and Alcoholism	182	3.04	National Institute of Mental Health	398	2.89
National Institute of Mental Health	42	2.03	National Institute of Mental Health	139	2.32	U.S. Department of Health and Human Services	354	2.57
National Institute on Alcohol Abuse and Alcoholism	34	1.64	National Institute of Neurological Disorders and Stroke	91	1.52	National Cancer Institute	206	1.49
National Heart, Lung, and Blood Institute	18	0.87	National Center for Research Resources	58	0.97	Canadian Institutes of Health Research	189	1.37
National Institute of Development Administration	18	0.87	National Cancer Institute	54	0.90	Medical Research Council	187	1.36
National Center for Research Resources	17	0.82	Medical Research Council	50	0.83	European Commission	183	1.33
U.S. Public Health Service	17	0.82	Deutsche Forschungsgemeinschaft	47	0.78	National Natural Science Foundation of China	179	1.30

Decade-by-Decade Breakdown by Most Productive Regions (1960s-2010s)

**1960-1969**			**1970-1979**			**1980-1989**		
**Region**	**# of Publications**	**% of Total**	**Country**	**# of Publications**	**% of Total**	**Region**	**# of Publications**	**% of Total**
United States	33	10.96	United States	979	41.68	United States	766	49.26
Israel	14	4.65	Canada	140	5.96	United Kingdom	84	5.40
Japan	13	4.32	United Kingdom	86	3.66	Canada	71	4.57
Germany	12	3.99	India	69	2.94	India	51	3.28
United Kingdom	10	3.32	Netherlands	47	2.00	Italy	42	2.70
Brazil	7	2.33	Germany	46	1.96	Japan	42	2.70
Canada	7	2.33	Brazil	44	1.87	Israel	38	2.44
India	6	1.99	Italy	39	1.66	Sweden	34	2.19
Italy	3	1.00	Sweden	37	1.58	Germany	32	2.06
Australia (Tied for Top 10)	2	0.66	Israel	34	1.45	Switzerland	28	1.80
Austria (Tied for Top 10)	2	0.66						
Czech Republic (Tied for Top 10)	2	0.66						
Russia (Tied for Top 10)	2	0.66						
Yugoslavia (Tied for Top 10)	2	0.66						
**1990-1999**			**2000-2009**			**2010-2019**		
**Region**	**# of Publications**	**% of Total**	**Region**	**# of Publications**	**% of Total**	**Region**	**# of Publications**	**% of Total**
United States	1016	49.06	United States	2237	37.31	United States	5818	42.20
United Kingdom	139	6.71	United Kingdom	623	10.39	United Kingdom	1068	7.75
Italy	102	4.93	Germany	439	7.32	Canada	1043	7.56
Japan	91	4.39	Italy	434	7.24	Germany	896	6.50
France	89	4.30	Spain	377	6.29	Italy	796	5.77
Spain	85	4.10	France	304	5.07	Australia	748	5.43
Germany	83	4.01	Australia	294	4.90	Spain	731	5.30
Israel	61	2.95	Canada	276	4.60	China	533	3.87
Canada	55	2.66	Netherlands	191	3.19	France	504	3.66
Australia	54	2.61	Japan	190	3.17	Netherlands	433	3.14

Decade-by-Decade Breakdown by Top 10 Languages of Publication (1960s-2010s)

1960-1969			1970-1979			1980-1989		
**Language**	**# of Publications**	**% of Total**	**Language**	**# of Publications**	**% of Total**	**Language**	**# of Publications**	**% of Total**
English	225	74.75	English	2085	88.76	English	1405	90.35
German	34	11.30	German	72	3.07	German	40	2.57
French	11	3.65	French	63	2.68	French	32	2.06
Japanese	9	2.99	Norwegian	28	1.19	Italian	16	1.03
Dutch	6	1.99	Spanish	27	1.15	Spanish	15	0.96
Italian	3	1.00	Italian	21	0.89	Swedish	10	0.64
Swedish	3	1.00	Swedish	13	0.55	Japanese	6	0.39
Danish	2	0.66	Dutch	10	0.43	Danish	5	0.32
Portuguese	2	0.66	Japanese	9	0.38	Dutch	5	0.32
Russian	2	0.66	Portuguese	6	0.26	Norwegian (Tied for Top 10)	3	0.19
						Polish (Tied for Top 10)	3	0.19

1990-1999			2000-2009			2010-2019		
**Language**	**# of Publications**	**% of Total**	**Language**	**# of Publications**	**% of Total**	**Language**	**# of Publications**	**% of Total**
English	1932	93.29	English	5433	90.63	English	13078	94.85
German	48	2.32	French	168	2.80	German	242	1.76
Spanish	19	0.92	German	132	2.20	Spanish	175	1.27
French	18	0.87	Spanish	100	1.67	French	169	1.23
Japanese	13	0.63	Dutch	34	0.57	Chinese	85	0.62
Dutch	9	0.43	Chinese	26	0.43	Portuguese	34	0.25
Italian	7	0.34	Portuguese	19	0.32	Polish	23	0.17
Portuguese	6	0.29	Czech	15	0.25	Dutch	21	0.15
Russian	4	0.19	Russian	13	0.22	Russian	17	0.12
Danish	3	0.14	Italian (Tied for Top 10)	12	0.20	Italian	15	0.11
			Japanese (Tied for Top 10)	12	0.20			
			Polish (Tied for Top 10)	12	0.20			
